# Understanding the Impact of Vaccination and Self-Defense Measures on Epidemic Dynamics Using an Embedded Optimization and Evolutionary Game Theory Methodology

**DOI:** 10.3390/vaccines11091421

**Published:** 2023-08-25

**Authors:** K. M. Ariful Kabir, MD Shahidul Islam, Mohammad Sharif Ullah

**Affiliations:** 1 Department of Mathematics, Bangladesh University of Engineering and Technology, Dhaka 1000, Bangladesh; 2 Department of Computer Science and Engineering, Green University of Bangladesh, Dhaka 1207, Bangladesh; shahidul@cse.green.edu.bd; 3 Department of Mathematics, Feni University, Feni 3900, Bangladesh; msu@feniuniversity.ac.bd

**Keywords:** optimal control, evolutionary game theory, vaccination, self-defense measure

## Abstract

Explaining how individual choice and government policy can appear in the same context in real society is one of the most challenging scientific problems. Controlling infectious diseases requires effective prevention and control measures, including vaccination and self-defense measures. In this context, optimal control strategies incorporating vaccination and self-defense measures have been proposed using the framework of evolutionary game theory. This approach accounts for individuals’ behavior and interactions in a population. It can provide insights into the effectiveness of different strategies for controlling the spread of infectious diseases. The optimal control strategy involves balancing the costs and benefits of vaccination, considering the dynamic interplay between the infected and susceptible populations. By combining evolutionary game theory with optimal control theory, we can identify the optimal allocation of resources for vaccination and self-defense measures, which can maximize the control of infectious diseases while minimizing costs. The model is utilized to analyze public health policies diseases, such as vaccination and self-defense strategies, to mitigate the spread of infectious in the context of delayed decision-making.

## 1. Introduction

Mathematical techniques for epidemic dynamics have assisted in understanding disease-spreading phenomena and have added significantly to the development of associated disease control strategies [1,2,3,4,5]. They are used to investigate disease patterns, disease forecasting, control strategies, intervention policies, and behavioral activities in the human population. Modeling and analyzing the complex relationship between disease incidence, control policies, and individuals’ behavior has become a challenging issue. With the increasing contagious disease of an epidemic, people may react with control and preventive behaviors such as volunteer vaccination, social distancing, mask-wearing, and stay-at-home policies that can reduce the risk of infection [6,7,8,9,10,11]. In this combined epidemic framework’s, the mean-field approximation method has been used, embedded with a vaccination strategy for an optimal control framework and protection strategy based on evolutionary game theory.

Most epidemic models of disease transmission are modifications of a basic model in biology known as SIR (susceptible–infected–recovered), partitioned into those susceptible to infection, infected, and removed or recovered from the disease [12]. These modifications include several control strategies and intervention policies that might affect the transmission of infectious diseases, such as vaccination, treatment, mask-wearing, social distancing, quarantine, and waning immunity [8,9,10,13,14]. To respond to some of these questions in the context of control strategy and preventive measures, we design a model with some feathers, as mentioned earlier, that emphasizes the vaccination and protection measures when individuals are prone to contagious diseases.

Optimal control theory is a branch of mathematics that can be employed widely in controlling and preventing the spread of contagious diseases by making decisions concerning intricate biological systems [15]. It is often applied in controlling the transmission of most diseases for which vaccine, awareness, treatment, or other provisions are available [7,8,10,11]. Zaman et al. [16] applied optimal control theory to an SIR model using vaccination as a control. The work carried out by Kirschner et al. [17] and Fister et al. [18] used optimal control methods for the treatment strategy in HIV cases and tumor cells under treatment for cancer. Also, Gaff and Schaefer [19] used optimal control theory for both vaccination and treatment to reduce infectious diseases. Recently, many optimal control models concerning epidemic diseases have been considered in the literature. They include time-delay SIRS epidemic dynamics [20], a tuberculosis model [21], an HIV model [22], a swine flu model [23], and a dengue fever [24] model by considering various types of control strategies. The fundamental concept behind optimal control in epidemiology is to explore the available strategies that effectively reduce the infection rate. In light of previous works, the current study aims to model an optimal control-based epidemic model for vaccination by considering the imperfectness of the vaccine. 

In response to an epidemic, human perception, decision, and behavior play a significant role in controlling disease spreading and imposing intervention policies. Several theoretical and experimental works have already been conducted on evolutionary game theory (EGT) to reveal human behavior in different respiratory diseases and intervention policies to reduce disease transmission [25,26,27]. Inspired by the various seasonal influenzas, several studies [11,28,29,30,31,32,33], which implemented several vaccination game dynamics, have analyzed the vaccine cost, effectiveness, and other factors for local and global time scales. The pioneering study of the dynamics of the behavioral game model for vaccination and mask-wearing in a single season introduced by Kabir et al. [10] revealed how individuals respond against vaccination and mask-wearing in epidemic spreading, respectively. Physical distancing can also be essential to delay or lessen the spread of disease [34,35]. Besides awareness [36], self-defense measures [37,38], quarantine, and isolation [11] have also received attention to alleviate the condition in the EGT framework. In social dilemma situations under EGT, individuals’ perceptions of self-protective measures can also effectively reduce contagious diseases [39,40,41]. In addition, most EGT-based epidemic models consider the inherent cost, benefit, and risk of infection to adopt the decision of intervention strategies [42,43,44,45]. As presented by Kabir et al. [10,46], the usual form of behavioral dynamics model on EGT to take self-protective measures has received attention. Following the above perception, here we model an epidemic self-protective game model considering maintaining self-protection costs and relative factors. Additionally, we develop a novel theoretical epidemic model that considers both optimal control theory for vaccination and the self-protective game model with the disease spreading over time. To the best of our knowledge, the proposed model is the first to confront two different controlling processes on the same framework to control epidemic spreading.

In this work, we deal with the issue of how to optimally combine the vaccination and self-protection measures such that the vaccine implementation works as an optimal control strategy and self-protection runs as a behavioral dynamic. It is important to mention here that optimal control is a process that is not an intelligent method; it is a straightforward deterministic method. On the other hand, an evolutionary game is an intelligent process that depends on the human decision mechanism. Optimal control can control disease; however, EGT has both control and anti-control tendency characteristics. The final goal of optimal control is optimizing the system to acquire maximum benefit; however, the goal of EGT is to find the Nash equilibrium. Note that this paper deals with both the optimal control strategy and the evolutionary game model by introducing the factors of vaccine efficacy, vaccine cost, and self-protection-maintained cost. This approach is different from the previous works, which concentrate on either the EGT model or the optimal control model, bringing more realistic models that incorporate both the evolutionary game theory (EGT) model and the optimal control model. By combining these two frameworks, we can better capture the complex dynamics of infectious disease transmission, including the behavioral responses of individuals to different mitigation policies, and optimize the allocation of resources to control the disease.

## 2. Model and Methods

**Figure 1:**
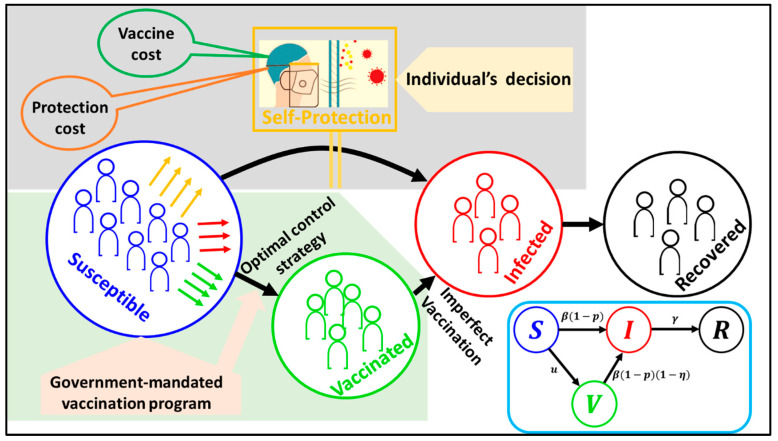
A schematic diagram of an epidemic model embedded in an optimal control strategy and evolutionary game theory is presented. The population is divided into four states: susceptible, vaccinated, infected, and recovered. This model is relevant for the epidemic season on a local time scale, implying that disease transmission, optimal control, and behavioral dynamics are happening simultaneously. The dynamic of the self-protection measure is influenced by the strategy-updating process on the framework of evolutionary game theory represented by the variable p. The parameter u demonstrates the vaccination-based optimal control strategy.

To begin our studies of the complete model, we consider a standard SIR (susceptible–infected–recovered) model for a population with a vaccine control strategy, vaccination compartment (V), and self-protection measure depicted in Figure 1. Model compartments include the expected number of susceptible (S), infected (I), vaccinated (V), and recovered states (R). The four-compartment model with the total population at time t is represented by N(t)=1; i.e., S(t)+I(t)+V(t)+R(t)=1. The fraction of suspected susceptible individuals is infected at a transmission rate β, and the fraction of vaccinated individuals become infected at a reduced rate (1−η)β, where η defines the vaccine efficacy. The control parameter u(t) determines the rate at which the susceptible are vaccinated in each time step, and the self-protection parameter p(t) is defined as a preferred rate of self-defense measure to avoid infection. In this model, the control parameter u(t) is set as a vaccine control strategy followed by the optimal control theory approach. However, the self-protection rate p(t) is considered for human behavioral dynamics in which an individual can choose to take self-defense measures or not (mask-wearing, handwashing, social distancing, etc.) in the framework of evolutionary game theory. Finally, infected individuals recover naturally at the rate γ. The dynamical equation of SVIR is given by

(1) $$\dot{S}(t)=-\beta \left(1-p\right)I(t)S(t)-uS(t),$$

(2) $$\dot{V}\left(t\right)=uS\left(t\right)-\beta (1-p)\left(1-\eta \right)I\left(t\right)V\left(t\right),$$

(3) $$\dot{I}\left(t\right)=\beta (1-p) [S\left(t\right)+\left(1-\eta \right)V\left(t\right)]I\left(t\right)-\gamma I\left(t\right),$$

(4) $$\dot{R}\left(t\right)=\gamma I\left(t\right).$$

### Derivation of Basic ${(R}_{0})$ and Effective ${(R}_{e})$ Reproduction Number

To proceed with our initial investigation, we will examine the methodology employed to calculate the basic reproduction number, denoted as $R_{0}$. This parameter has substantial implications in the field of epidemiological modeling, as it has been demonstrated to assist in comprehending the conditions of stability. If the value of this numerical quantity is less than one, it is plausible to expect a state of stability. Conversely, if the value exceeds one, it is conceivable that a state of instability may arise. In this scenario, we will employ the next-generation matrix approach to achieve the reproductive value, as outlined below:

$$R_{0}=\frac{\beta (1-p)(1+\left(1-\eta \right))}{\gamma },$$

The expected effective reproduction number denoted by $R_{e}$ is,

$$R_{e}=\frac{\beta (1-p)}{\gamma }S\left(t\right)+\frac{\beta (1-p)\left(1-\eta \right)}{\gamma }V\left(t\right).$$

Here, the obtained effective reproduction number is $R_{e}=\frac{\beta (1-p) [S\left(t\right)+(1-\eta )V\left(t\right)]}{\gamma }$. The following proposition can decide the existence of the stability of the proposed epidemic dynamic: (i) if *Re* > 1, then the disease-free equilibrium (DFE) is unstable, and (ii) for *Re* < 1, the DFE is stable.

## 3. Statement of the Optimal Control Problem

To compute the optimal vaccination strategy that would minimize infected individuals and minimize the vaccine factors, we consider the optimal control problem [47,48,49,50] as

(5) $$\underset{u\in U}{\min}J\left(u\left(t\right)\right)=\int_{0}^{T}\left(\frac{A}{2}u^{2}\left(t\right)+BS\left(t\right)\right)dt$$

Equations (1)–(4) and belonging to a set of acceptable controls, $U=\left\{u:u_{min}\leq u\left(t\right)\leq u_{max}\right\},$ where, $0\leq u_{min}<u_{max}\leq 1$. The term A represents the weighted costs involved with using control u(t) and B defined to denote the weight coefficient of the susceptible individuals [47,48,49,50].

To characterize the optimal control problem, we consider the Hamiltonian H to apply the Pontryagin’s maximum principle. For simplicity, we take $x\left(t\right)={ [S\left(t\right),V\left(t\right),I\left(t\right),R(t)]}^{T}$, $\lambda \left(t\right)={ [\lambda_{1}\left(t\right),\lambda_{2}\left(t\right),\lambda_{3}\left(t\right),\lambda_{4}(t)]}^{T}$ and $u\left(t\right)$ given the Hamiltonian

(6) $$H=\frac{A}{2}u^{2}\left(t\right)+BS\left(t\right)+\lambda_{1}\left(t\right) [-\beta (1-p)IS-uS]+\lambda_{2}\left(t\right) [uS-\beta (1-p)(1-\eta )IV]\;+\lambda_{3}\left(t\right) [\beta (1-p)IS+\beta (1-p)\left(1-\eta \right)IV-\gamma I]+\lambda_{4}\left(t\right)\gamma I.$$

According to state Equations (1)–(4), the co-state equations are

(7) $$\lambda_{1}^{\prime }=-\frac{\partial H}{\partial S}=-B+\lambda_{1} [\beta \left(1-p\right)I+u]-\lambda_{2}u-\lambda_{3}[\beta \left(1-p\right)I],\;\lambda_{2}^{\prime }=-\frac{\partial H}{\partial V}=\lambda_{2}\beta (1-p)\left(1-\eta \right)I-\lambda_{3}\beta \left(1-p\right)\left(1-\eta \right)I,\;\lambda_{3}^{\prime }=-\frac{\partial H}{\partial I}=\lambda_{1}\beta \left(1-p\right)S+\lambda_{2}\beta \left(1-p\right)\left(1-\eta \right)V-\lambda_{3} [\beta \left(1-p\right)S+\beta \left(1-p\right)\left(1-\eta \right)V-\gamma ]-\lambda_{4}\left(t\right)\gamma \;\lambda_{4}^{\prime }=-\frac{\partial H}{\partial R}=0.$$

With transversality conditions

$$\lambda_{i}\left(T_{f}\right)=0,\;\;i=1,2,3,4$$

Further, the optimal control is given by

$$u_{1}^{*}\left(t\right)=min\left(max\left(0,\frac{1}{A}\left(\lambda_{1}\left(t\right)-\lambda_{2}\left(t\right)\right)S(t)\right),1\right)$$

Also,

(8) $$\frac{\partial H}{\partial u}=Au-\lambda_{1}S+\lambda_{2}S=0\;u=\frac{(\lambda_{1}-\lambda_{2})S}{A}.$$

Here, the transversality conditions [47,48,49,50] are $\lambda_{1}=1,\lambda_{2}=1,\lambda_{3}=1$ and $\lambda_{4}=-1$. The Hamiltonian function *H* and u is strongly convex. Also, the right-hand side of the state and co-state equations are Lipshitz continuous.

## 4. Evolutionary Dynamics

In current behavioral dynamics under EGT, each participant in the susceptible group can choose whether to take self-protection/self-defense measures against diseases or not, depending on the disease incidence, individual socioeconomic conditions, and vaccine status. Individuals who maintain self-defense measures have the self-defense measure cost provided by individuals, denoted by $C_{P}$. People who already participate or intend to participate in the government’s vaccine program also have a charge for vaccination, characterized by vaccination cost, $C_{V}$. If p denotes the protection rate that reduces the disease transmission, the equation that presents the behavioral evolutionary dynamics is

(9) $$\frac{dp}{dt}=mp\left(1-p\right) [-\left(1-C_{V}\right)V-\omega C_{P}+C_{I}\int_{0}^{t}I\left(\tau \right)d\tau ].$$

where $C_{I}$ is the cost for infection $\left(C_{I}=1\right)$, and the term m is defined as the transferred parameter to transfer an individual’s fraction to the disease-transmission-reduction rate. Also, ω is used as a controlling parameter of self-protection measures.

To incorporate the concept of the fraction of self-defense measures (FSD) into an epidemic model integrated with evolutionary game theory (EGT), we examine the proportion of individuals who avail themselves of self-defense protection. The calculation of FSD involves several steps. Initially, we determine the final epidemic size (FES) without any policy for self-defense measures, reaching an equilibrium state. Subsequently, we analyze the behavioral dynamics based on evolutionary game theory, considering the presence of self-defense strategies, and calculate the system’s Nash equilibrium (NE).

## 5. Result and Discussion

The fundamental purpose of this study is to embed two control mechanisms, evolutionary game theory and control strategy, between two interventions, self-defense measures and vaccination, respectively, for epidemic control. In this section, we describe experiments that were conducted to investigate the impact of optimal control theory and evolutionary game theory on the spread of the epidemic while considering the scenario where susceptible individuals either participate in a vaccine program or take self-defense measures. Vaccine efficacy, vaccine cost, and self-defense-maintained cost are used to analyze the disease incidence and epidemic control to obtain the best control strategy. To obtain the governing equation’s time series graphs and trajectory, we use the finite difference method to numerically simulate the coupled optimal control strategy and evolutionary game theory for an infinite and well-mixed population on a local time scale. 

**Figure 2:**
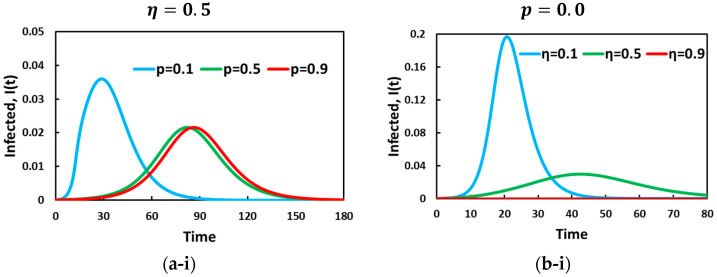

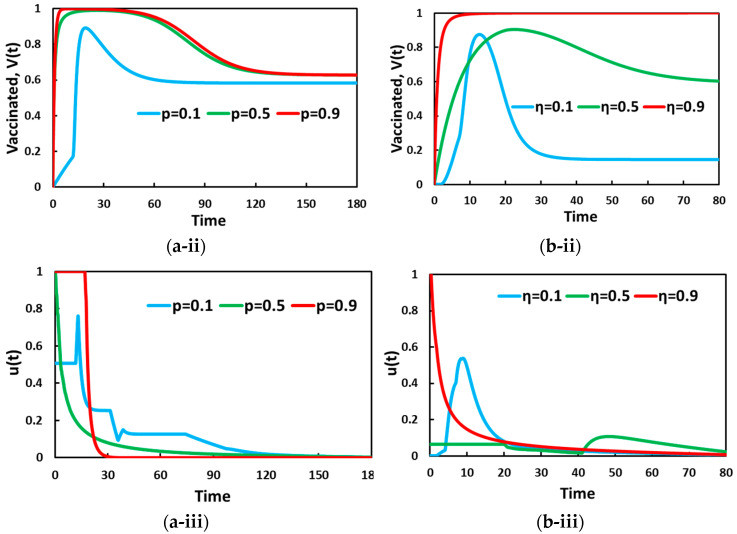
Effect of vaccination-based optimal control aspect on (a-*) infected, (b-*) vaccinated, and (c-*) u(t) along the time scale by considering an analysis without evolutionary game dynamics. In sub-panel (*-i), in which vaccine efficacy η=0.5, the solid blue, green, and red lines represent the case (no provisions) for p=0.1, p=0.5, and p=0.9, respectively. On the other hand, in sub-panel (*-ii) for p=0.0, the solid blue, green, and red lines represent η=0.1, η=0.5, and η=0.9, respectively. The other related parameters are β=0.83333, and γ=0.3333.

Figure 2 illustrates the portion of infected (a-*), vaccinated (b-*), and (c-*) u(t) individuals for self-defense measures and the optimal control strategy aspect over time. Sub-panel (*-i) displays the fraction of infected and vaccinated individuals for the vaccination strategy over varying constant self-protection parameters p=0.1, 0.5, and 0.9 with fixed vaccine efficacy η=0.5. In the absence of behavioral dynamics, sub-panel (*-ii) presents the disease incidence and vaccination uptake for vaccine efficacy, η=0.1, 0.5, and 0.9, when the self-protection rate is p=0.0. Sub-panel (a-i) shows that the fraction of infected individuals decreases as the self-defense measure increases (p=0.9). Interestingly, our results also explore how the higher personal protection measures can flatten the curve and can be seen as an operative tool to delay the epidemic peak. Further, the sub-panel (b-i) observation illustrates that vaccinated individuals are enhanced as the personal protection measure rate increases. To understand sub-panel (b-i), we can refer to sub-panel (a-i), in which the time-delay effect resulting from self-defense measures enhances vaccination uptake. As p increases, the disease transmission rate decreases, increasing susceptible individuals, which ensures that there are highly vaccinated individuals in the community. Thus, the higher the rate of the self-defense measure, the more people can avoid infection, in which most people participate vaccination programs, and consequently, the number of infected individuals is reduced. In panel (-*iii), the control variable for the vaccine strategy demonstrates a pattern of change. The objective is to maintain the maximum control force starting from the first day and continue until a state of equilibrium is reached, at which point the control force is gradually reduced to zero. The control variable, represented by u(t), refers to the manipulative actions taken in the context of the vaccine strategy. It involves determining the intensity or magnitude of the control force applied to influence the outcome of the process. From the initial day of implementation, the control force is set at its maximum level, indicating a proactive approach to address the target point. This strong control force signifies a heightened effort to drive the desired outcome and maximize the effectiveness of the vaccine strategy.

Now, in sub-panel (a-ii) and (b-ii), the impact of vaccine efficacy for η=0.1, 0.5, and 0.9 is depicted in presenting the infected and vaccinated individuals in the absence of a self-defense measure (p=0.0). Throughout, we can realize that higher values of vaccine reliability present a lower fraction of infected individuals that can be justified by the vaccinated individuals in sub-panel (b-ii). Interestingly, the infected line (red and green) shows an entirely waving tendency for more inferior η. One possible reason behind this could be justified by the lower vaccine efficacy enforced by the optimal control strategy. According to the vaccination control strategy, individuals initially participate in the vaccine program; however, due to the imperfectness of vaccination, individuals are infected. 

**Figure 3:**
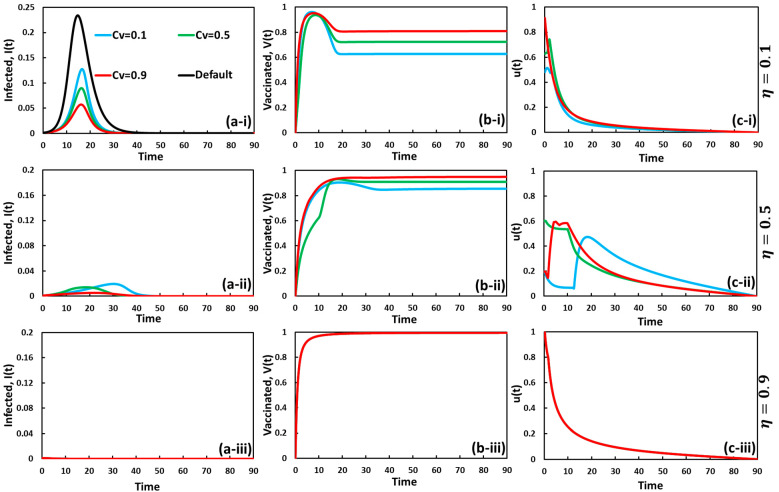
Dynamic behavior of a system comprising the optimal control strategy and vaccination game model under the influence of self-defense measure cost $C_{P}=0.0$ over the time series. The cases of (a-*), (b-*), and (c-*) for infected, vaccinated, and u(t) with $C_{V}=0.1$ (blue), $C_{V}=0.5$ (green), and $C_{V}=0.9$ (red) are considered here. The solid black line represents the setting referring to the no provision (without optimal control and without-vaccination game theory). Sub-panels (-*i), (-*ii), and (-*iii) represent, for vaccine efficacy, η=0.1, η=0.5, and η=0.9, respectively. The other related parameters are β=0.83333 and γ=0.3333.

**Figure 4:**
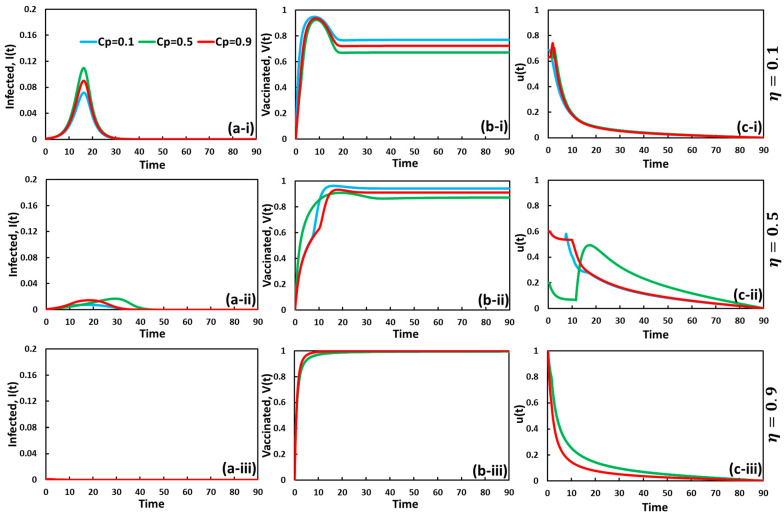
Dynamic behavior of a system comprising the optimal control strategy and vaccination game model under the influence of vaccination cost $C_{V}=0.001$ over the time series. The cases of (a-*), (b-*), and (c-*) for infected, vaccinated, and u(t) with $C_{P}=0.1$ (blue), $C_{P}=0.5$ (green), and $C_{P}=0.9$ (red) are considered here. Sub-panels (*-i), (*-ii), and (*-iii) represent, for vaccine efficacy, η=0.1, η=0.5, and η=0.9, respectively. The other related parameters are β=0.83333 and γ=0.3333.

To explore an insightful understanding of evolutionary game theory and optimal control theory in the same context of the two interventions, self-defense measure and vaccination, we present line graphs of the fraction of infected and vaccinated individuals in Figure 3 and Figure 4. Here, Figure 3 and Figure 4 display the set of line graphs for $C_{P}=0.0$ and $C_{V}=0.0$, respectively, in which, in each figure, panel (a-*), panel (b-*), and panel (c-*) present the fraction of infected, vaccinated, and *u*(*t*) over time for varying vaccine efficacy as (*-i) η=0.1, (*-ii) η=0.5, and (*-iii) η=0.9, respectively.

In Figure 3a-*, the fraction of infected individuals declined as vaccine reliability (eta) increased. Since vaccine reliability quantifies the enhancement of immunized people in society, it was expected to significantly control disease outbreaks. It was also observed that the infected outcomes were higher when η=0.1 (sub-panel (a-i)), and an almost disease-free equilibrium state was obtained for η=0.9 (sub-panel (a-i)). The introduction of control policies (vaccination) can successfully perform better when vaccine efficacy is high and the cost is low, as displayed in sub-panel (a-iii) and (b-iii). The autonomous vaccination process works effectively and does not involve behavioral dynamics (self-defense) when its reliability performs extraordinarily; higher vaccinated areas ensure more efficient ways to reduce the contagious disease severity. 

An in-depth analysis of the progression of vaccination costs identifies some exciting phenomena that need to be pointed out. Firstly, we can see from sub-panel (a-i) that the higher vaccination cost more effectively reduces diseases than the lower cost when $C_{V}=0.1$ and η=0.1. That means that spreading the disease can be controlled by introducing the self-defense measure strategy free of charge. In such a situation, the vaccine control policy is less effective than self-defense. Thus, results from dual-policy cases are more influenced by the self-defense policy than by vaccination. When the self-defense strategy is working very well and the vaccine loses reliability due to lower η, then the vaccine cost plays the opposite role in reducing disease transmission, where increasing the cost decreases infection. Apart from self-defense measures involving human decision making, the health policymaker must ensure higher efficacy of vaccination to address the pressing issue. Overall, the changing pattern in panel (c-*) illustrates a strategic and adaptive approach to the control variable in the vaccine strategy. It emphasizes the initial maximum control force to drive early progress. Subsequently, it transitions to a reduced control force as the system approaches equilibrium, ensuring a balanced and optimized strategy implementation.

We observe another setting of investigation showing the variation in self-defense cost for the vaccination cost, $C_{V}=0.5$, in Figure 4. All sub-panels suggest that the sensitivity mostly comes from self-defense costs. In general, the lower self-defense costs reduced infections and boosted vaccination. The self-protected individuals who stayed in the susceptible state are more likely to be vaccinated. This is why the lower cost of self-defense enhances vaccination coverage, meaning that one can control the epidemic transmission by staying up on susceptible states via self-defense measures. Therefore, results from the dual policy cases are influenced mainly by the self-defense strategy to slow disease transmission, which represents the time-delay effect. On the other hand, vaccination with higher reliability effectively permanently reduces diseases when the vaccine cost is low. Thus, double-control approaches (self-defense and vaccination) are the most effective way to reduce the severity of contagious disease. 

**Figure 5:**
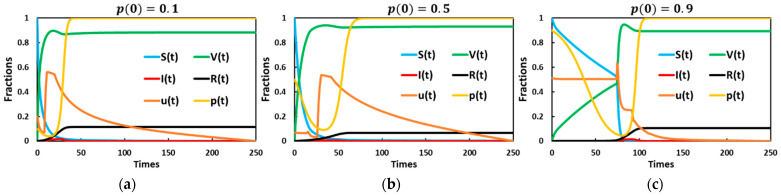

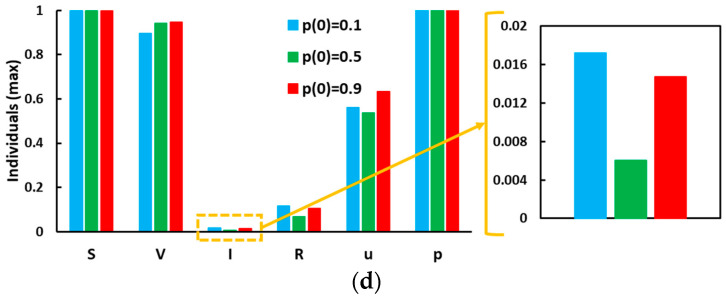
Dynamic behavior of a system comprising an optimal control strategy and vaccination game model under the influence of an initial self-defense awareness rate, (**a**) p(0)=0.1, (**b**) p(0)=0.5, and (**c**) p(0)=0.9, over time. In case (**d**), the distribution bar graph presents the maximum fraction of individuals for three cases in which infected individuals are shown as an insight figure.

Figure 5 illustrates the impact of the initial self-protection measure on the optimal control strategy’s vaccination uptake. It highlights how the presence of initially self-protected individuals influences the timing of infection onset and the rate at which vaccination uptake peaks. In Figure 5a, when the initial self-protection measure rate is lower, infections arise early, resulting in the rapid spread of the disease. Consequently, vaccination uptake also reaches its peak within a very short period, indicating that a refusal or lack of adherence to self-protection measures accelerates the onset of infections, leading to a faster progression of the epidemic. Simultaneously, individuals recognize the urgency and promptly seek vaccination, contributing to a swift increase in vaccination rates. However, in Figure 5c, when the proportion of initially self-protected individuals is higher, there is a delay in the spread of the disease. A more significant number of individuals practicing self-protection measures slows the transmission of the infection, effectively slowing down the epidemic. This delay in spreading conditions also affects the rate at which vaccination uptake occurs. The slower progression of the epidemic allows more time for individuals and governments to make informed decisions regarding vaccination strategies and other necessary interventions. Therefore, higher initial self-protection rates reduce the incidence of infection and delay the onset of an epidemic. This delay provides valuable time for authorities to implement appropriate measures, allocate resources, and effectively manage the situation. Moreover, the slower pace of vaccination uptake allows for a more strategic and organized approach to immunization, ensuring that it is carried out efficiently and reaches a more significant portion of the population.

A notable observation can be made from Figure 5b, which examines the scenario when the initial self-protection rate is intermediate (p(0)=0.5); it becomes apparent that a lower infection rate is observed. In Figure 5d, the distribution bar graph provides insights into the maximum number of individuals in various compartments. In this case, S (susceptible individuals) and p (proportion of self-protected individuals) show no significant differences across the three scenarios. However, it is worth noting that V (vaccinated individuals) and u (control variable) exhibit higher values when the initial self-protection rate is set to p(0)=0.9. On the other hand, both I (infected individuals) and R (recovered individuals) display a noticeable reduction when the initial self-protection rate is at its intermediate value of p(0)=0.5. From these observations, implementing vaccination and self-protection measures at a reasonable rate reduces infection rates and ultimately decreases the final size of the epidemic. The scenario with an intermediate initial self-protection rate of p(0)=0.5 demonstrates a favorable outcome, showing lower infection rates than the other cases. Therefore, the findings from Figure 5b and d highlight the importance of balancing vaccination and self-protection measures. Implementing these measures at a reasonable and coordinated rate can effectively mitigate the spread of infections, reducing the overall impact of the epidemic.

**Figure 6:**
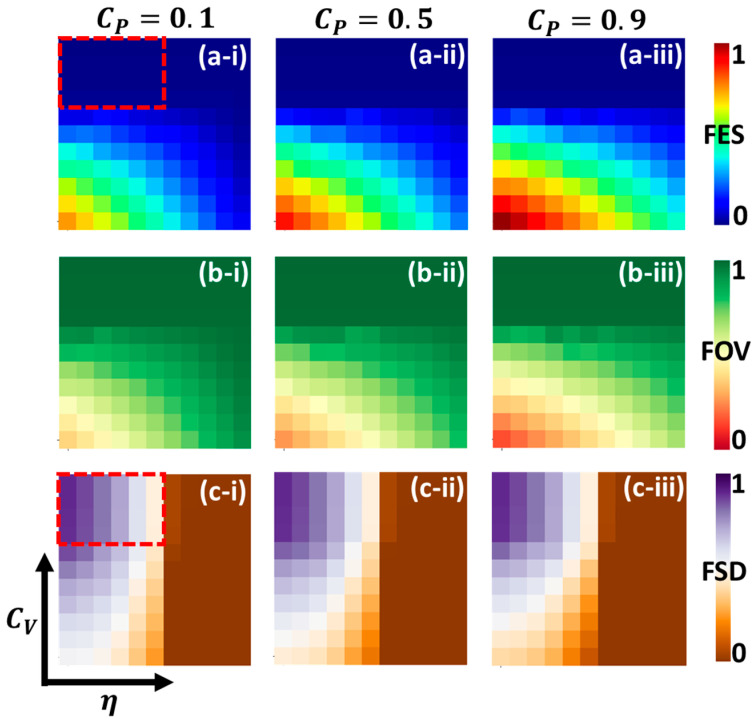
Two-dimensional phase diagram representing, for (a-*), final epidemic size; for (b-*), fraction of vaccinators; and for (c-*), the fraction of self-defense measured individuals over vaccine effectiveness (η) and vaccination cost ($C_{V}$). Here, panels (*-i), (*-ii), and (*-iii) show, for the various self-protection cost, $C_{P}=0.1$, 0.5, and 0.9. Other values are as follows: β=0.83333, γ=0.3333, A=0.1, and B=1.

Figure 6 displays multiple graphs in panels (a-*), (b-*), and (c-*) to emphasize the 2D phase diagrams. These diagrams focus on the final epidemic size (FES), the fraction of vaccinators (FOV), and the fraction of self-protected individuals (FSD) within the parameter plane defined by ($\eta ,C_{V}$). The sub-panels (-i), (-ii), and (*-iii) provide detailed representations of the overall phase behavior for different self-protection costs ($C_{P}$), specifically $C_{P}=0.1,\;0.5,$ and 0.9. Notably, all the 2D graphs in the figure illustrate the disease-free equilibrium region, visually represented by the color blue.

In panel (*-i), when the cost of self-defense measures ($C_{P}$) is relatively low ($C_{P}=0.1$), these measures significantly contribute to reducing infections compared to when $C_{P}$ is higher. In this scenario, a more effective vaccine leads to a lower final epidemic size (FES) and a higher fraction of vaccinators (FOV). However, considering the vaccine cost ($C_{V}$), an exciting observation arises. A disease-free equilibrium is observed for higher $C_{V}$ values, primarily driven by the adoption of self-defense measures (FSD), represented by a rectangular red box in panels (a-i) and (c-i). When the cost of vaccination is high, people are less inclined to take the vaccine and instead rely on self-defense measures. Moving on to panels (*-ii) and (*-iii), where $C_{P}$ is increased, we see that the contribution of self-defense measures (FSD) decreases and the final epidemic size (FES) increases. Another noteworthy phenomenon is the decrease in the fraction of vaccinators (FOV) as $C_{P}$ increases, attributed to the delayed effect of self-protection measures. As discussed earlier, self-defense measures delay the spread of the disease. Further, if the cost of participating in self-protection measures is very high, people are less likely to engage in them. As a result, the disease burden increases while vaccination rates decrease. Thus, when the cost of self-defense measures is low, and they significantly reduce the number of infections. However, with higher prices, people prioritize vaccination, leading to a disease-free equilibrium. Increasing the cost of self-defense reduces vaccinated individuals and leads to higher infection rates. Additionally, the high cost of self-protection measures discourages their adoption, increasing disease burden and decreasing vaccination rates.

**Figure 7:**
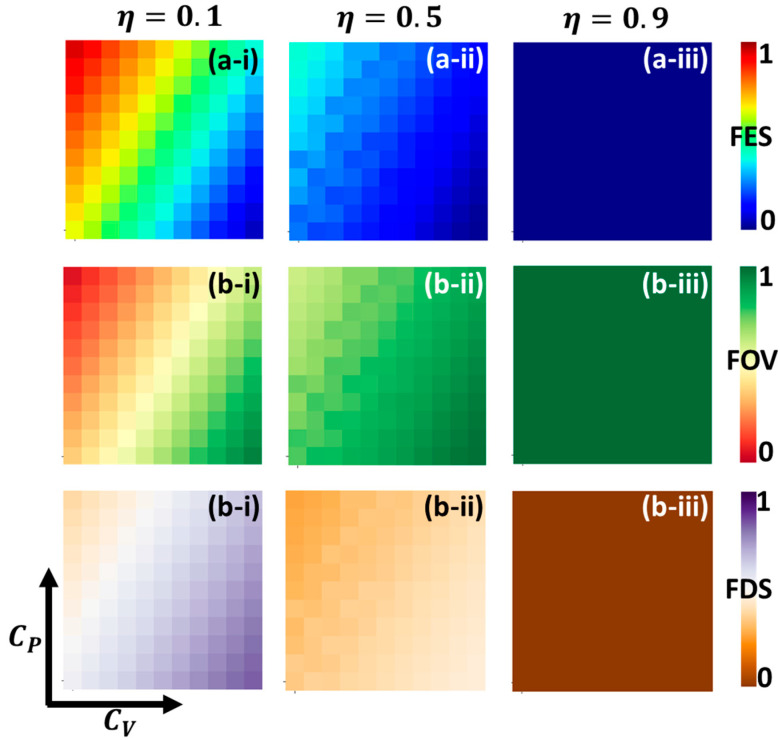
Two-dimensional phase diagram representing, for (a-*), final epidemic size; for (b-*), fraction of vaccinators; and for (c-*), fraction of self-defense measured in individuals over the vaccination cost ($C_{V}$ ) and self-protection cost $\left(C_{P}\right)$. Here, panels (*-i), (*-ii), and (*-iii) show, for the various self-protection costs, $C_{P}=0.1$, 0.5, and 0.9. Other values are as follows: β=0.83333, γ=0.3333 , A=0.1, and B=1.

Figure 7 showcases several graphs in panels (a-*), (b-*), and (c-*) that highlight the 2D phase diagrams. These diagrams are focused on capturing the final epidemic size (FES), the fraction of vaccinators (FOV), and the fraction of self-protected individuals (FSD) within the parameter space defined by ($C_{V},C_{P}$). Sub-panels (*-i), (*-ii), and (*-iii) present detailed representations of the overall phase behavior under different vaccine effectiveness values (η), specifically η=0.1, 0.5, and 0.9. 

In Figure 7, it can be seen that increasing vaccine effectiveness controls infections, as depicted in panel (*-iii) from FOV. The self-defense measure could be more attractive and reasonable for most individuals if the vaccine effectiveness is lower (panels (*-i)); they cannot rely on the vaccine policy to protect themselves against the infection. The trend of infected individuals seems opposite, along with vaccine cost and self-defense measure cost, which indicates an influence driven by decision-based self-defense measures and authority-based vaccination policy. Thus, the contribution shown by self-protection and vaccination enormously benefits everyone in society. One obvious notable point with self-defense measures is that when reasonable self-defense measures are obtained at a lower price, they encourage people to take self-protection, significantly enhancing government-imposed vaccination programs. 

## 6. Conclusions

The decision to impose government-mandated vaccination versus relying on individual self-defense measures is a complex issue that requires consideration of various factors such as vaccine cost, vaccine efficacy, and self-defense measure cost. While vaccines have proven to be highly effective in preventing the spread of infectious diseases, the cost of implementing and distributing vaccines can be a significant barrier, particularly in low-income countries. Moreover, vaccine hesitancy and misinformation can also undermine the success of vaccination programs. On the other hand, while effective, self-defense measures such as social distancing, wearing masks, and hand hygiene can be burdensome and inconvenient for individuals, and their effectiveness depends on widespread adoption.

The integration of evolutionary game theory and optimal control theory offers a promising framework for analyzing the efficacy of vaccination and self-defense measures in controlling infectious diseases. Our findings suggest that effective control strategies must balance the costs and benefits of these measures, considering the dynamic interplay between the infected and susceptible populations. Furthermore, by incorporating the delayed effects of mitigation policies, our approach can provide more realistic predictions of disease-transmission dynamics and the effectiveness of different control strategies.

Overall, the combination of evolutionary game theory and optimal control theory provides a robust framework for modeling and analyzing the complex dynamics of infectious disease transmission and designing effective control strategies that can be adapted to different scenarios and contexts. In many cases, a combination of both government-mandated vaccination and self-defense measures may be the most effective way to control the spread of infectious diseases. Governments must also prioritize efforts to improve vaccine education and access to ensure that everyone has the opportunity to protect themselves and their communities from infectious diseases.

## Data Availability

The datasets generated and/or analyzed during the current study are not publicly available due to the original code but are available from the corresponding author on reasonable request.
